# Antidepressant effect of bright light therapy on patients with Alzheimer’s disease and their caregivers

**DOI:** 10.3389/fphar.2023.1235406

**Published:** 2023-11-15

**Authors:** Xi Mei, Chenjun Zou, Zizhen Si, Ting Xu, Jun Hu, Xiangping Wu, Chengying Zheng

**Affiliations:** ^1^ Key Lab, Ningbo Kangning Hospital, Ningbo, Zhejiang, China; ^2^ Department of Geriatric, Ningbo Kangning Hospital, Ningbo, Zhejiang, China; ^3^ Medical College, Ningbo University, Ningbo, Zhejiang, China

**Keywords:** bright light therapy, Alzheimer’s disease, depression, EEG, biomarkers

## Abstract

**Background:** As a non-pharmacologic treatment, bright light therapy (BLT) is often used to improve affective disorders and memory function. In this study, we aimed to determine the effect of BLT on depression and electrophysiological features of the brain in patients with Alzheimer’s disease (AD) and their caregivers using a light-emitting diode device of 14000 lux.

**Methods:** A 4-week case-control trial was conducted. Neuropsychiatric and electroencephalogram (EEG) examination were evaluated at baseline and after 4 weeks. EEG power in delta (1–4 Hz), theta (4–8 Hz), alpha (8–12 Hz), and beta (12–30 Hz) bands was calculated for our main analysis. Demographic and clinical variables were analyzed using Student’s t test and the chi-square test. Pearson’s correlation was used to determine the correlation between electrophysiological features, blood biochemical indicators, and cognitive assessment scale scores.

**Results:** In this study, 22 in-patients with AD and 23 caregivers were recruited. After BLT, the Hamilton depression scale score decreased in the fourth week. Compared with the age-matched controls of their caregivers, a higher spectral power at the lower delta and theta frequencies was observed in the AD group. After BLT, the EEG power of the delta and theta frequencies in the AD group decreased. No change was observed in blood amyloid concentrations before and after BLT.

**Conclusion:** In conclusion, a 4-week course of BLT significantly suppressed depression in patients with AD and their caregivers. Moreover, changes in EEG power were also significant in both groups.

## Introduction

Alzheimer’s disease (AD) is a progressive degenerative disease affecting cognitive functions and mental health. Its clinical manifestations include memory impairment and additional cognitive domain impairments in executive functions, attention, language, social cognition and judgment, psychomotor speed, and visuoperceptual or visuospatial abilities (Masters et al., 2015; Scheltens et al., 2016; Scheltens et al., 2021). Cognitive impairment is considered a transitional stage between normal aging and AD. China has a high prevalence of dementia and cognitive impairment (Jia et al., 2020).

AD may be accompanied by mental and behavioral symptoms, such as depressed mood and apathy in the initial stages, and also by psychotic symptoms, irritability, aggression, confusion, gait and mobility abnormalities, and seizures in the later stages (Altomari et al., 2022). In the long-term care process, caregivers of patients with AD may also suffer from mental health problems (Corrêa et al., 2019; Carbone et al., 2021). In previous studies, depression occurred in 50% of patients with AD, thereby increasing the caregivers’ burden (Chi et al., 2014). Symptoms of depression can precede a clinical diagnosis of AD for years or occur around the onset of AD (Diniz et al., 2013). Caring for a loved one with AD can increase the risk of depression in caregivers, and symptoms of depression typically persist over time (Liu et al., 2017; Chai et al., 2018).

Aside from medication, a number of non-pharmacological treatments can be used to improve a patient’s health condition. According to previous studies, exposure to bright light may improve sleep and ease depression and agitation in people with AD (Peter-Derex et al., 2015; van Maanen et al., 2016; Roccaro et al., 2020). Persons living with AD or vascular dementia who were exposed to bright light therapy (BLT) demonstrated significantly improved scores on the Mini-Mental State Examination (MMSE) scale, compared to exposure to dim light therapy (Lu et al., 2023). BLT has also been used for decades to treat nonseasonal depression and other mood disorders (Al-Karawi and Jubair, 2016; Wang et al., 2020).

Regardless of clinical manifestations, AD biomarkers, including amyloidosis, tauopathy, and neurodegeneration, are important for diagnosis and to evaluate the effectiveness of therapy in this disease (Jack et al., 2018). Electroencephalogram (EEG) biomarkers can be used to reflect the effects of AD neuropathology on functional brain networks (Pfurtscheller and Lopes da Silva, 1999). Because of its high temporal resolution, we can investigate EEG rhythms at different frequency bands during a resting-state condition in patients with cognitive decline (Caravaglios et al., 2023). Although changes in electrophysiological features are not specific for patients with AD, compared to older adults without cognitive impairment, patients with AD or dementia with mild cognitive impairment were characterized by changes in EEG rhythms during resting-state condition (Babiloni et al., 2020).

This study aimed to determine the effect of BLT on depression and electrophysiological features of the brain in patients with AD and their caregivers using a light-emitting diode device with an intensity of 14000 lux.

## Methods

### Participants

We recruited inpatients and their caregivers form Geriatric Center of Ningbo Kangning Hospital between September 2022 and March 2023. Potential participants were recommended to our experienced research psychiatrists for further study. Ultimately, 22 patients with AD and 23 caregivers were recruited in this study. The patients with AD were diagnosed using the Diagnostic and Statistical Manual of Mental Disorders, fifth edition criteria (First, 2013). All patients met the following inclusion and exclusion criteria: 1) diagnosed with AD by two research psychiatrists; 2) provision of informed consent; 3) disease course >3 months; 4) cholinesterase inhibitor (donepezil) and non-competitive N-methyl-d-aspartate receptor antagonist (memantine) use; 5) no history of other mental illnesses, including schizophrenia and delirium; and 6) no physical diseases.

### Neuropsychiatric evaluation

The neuropsychological evaluation of cognition was confirmed by MMSE scores <17, 20, and 24 in people with no, primary school, and junior high school education, respectively (Li et al., 2016). The AD assessment scale—cognitive subscale (ADAScog) was used to evaluate patients’ memory, language, and other cognitive impairments (Rosen et al., 1984). The Hamilton depression scale (HAMD) was adopted to evaluate depression in patients and their caregivers (Zimmerman et al., 2013).

### Experimental protocol

The light therapy equipment used for treatment was designed by the Geriatric Center of Ningbo Kangning Hospital as described in our previous study (Zou et al., 2022). Light therapy with peak strength of 14000 lux was conducted from 9:00 a.m. to 9:30 a.m. each day for 4 weeks. The patients and their caregivers sat in front of the light therapy device with their eyes open to allow the light to reach the retinas for BLT. The distance between the participants and light source was 50 cm.

### EEG examination and analysis

The participants were seated in a comfortable chair. A 128-channel EEG (EGI System 300; Electrical Geodesic Inc., Eugene, OR, United States) configured in the standard 10–20 montage was recorded with reference to linked mastoids. The sampling rate was 500 Hz using an amplifier with low and high cutoff frequencies of 0.1 and 100 Hz, respectively.

EEG recordings were compiled during a resting state using a structured testing and acquisition software platform (5 min with the eyes opened and 5 min with the eyes closed). During the open-eye task, participants were instructed to stare directly at a black fixation cross located in the center of a gray background. During the closed-eye task, they were instructed to close their eyes while maintaining wakefulness. NetStation software (Electrical Geodesic Inc., OR, United States) was used for recording. The impedance for all electrodes was kept below 50 kΩ. Offline data analysis was conducted with the open-source EEGLAB toolbox.

### Blood enzyme-linked immunosorbent assay

Blood samples were collected before breakfast using a winged blood collection set. Approximately 5 mL of whole blood was collected in a procoagulant tube. To allow the measurement of plasma peptides, blood was immediately centrifuged at 1,400 rpm using a BY-600A type medical centrifuge (Beijing Baiyang Medical Devices Co., Beijing, China) for 10 min. All blood samples were processed within 30 min of collection and immediately frozen at −80°C. Blood samples were thawed immediately before analysis.

Serum amyloid-β (Aβ), Aβ_40_, Aβ_42_, interleukin (IL)-1β, and melatonin (MT) levels were estimated using enzyme-linked immunosorbent assay kits (Shanghai Yuanye Bio-Technology Co., Shanghai, China). All procedures were performed according to the manufacturer’s instructions. Absorbance was measured at 450 nm using a Sunrise-basic enzyme labeling instrument (Tecan Group Ltd., Mannedorf, Switzerland) with a reference wavelength of 690 nm. These measurements were transformed into concentrations by comparing the optical densities of the samples with the standard curve values.

### Statistical analysis

Data are presented as the mean ± standard deviation (SD). Demographic and clinical variables were compared and analyzed between the different groups using Student’s t test for continuous variables and the chi-square test for categorical variables. Pearson’s correlation was used to determine the correlation among electrophysiological features, blood indicators, and cognitive assessment scale scores. Statistical significance was set at *p* < 0.05. Statistical Package for the Social Sciences (SPSS version 19.0, IBM Corp., Armonk, NY, United States) was used for all analyses.

## Results

### Clinical assessment

**FIGURE 1:**
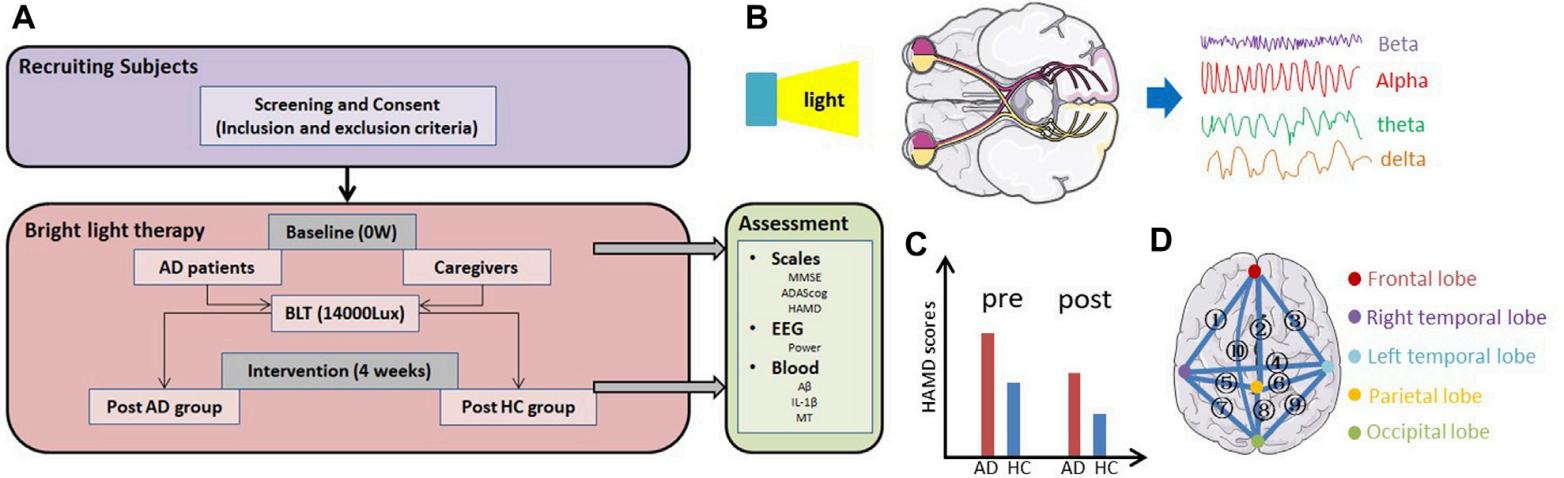
Overview of the study. **(A)** Protocol for bright light therapy in patients with AD and their caregivers (HC). **(B)** Light therapy modulated the EEG signal through the retina to the cortex. **(C)** HAMD scores, which reflect the level of depression in participants, may be change before and after therapy. **(D)** ① to ⑩ represent the relationship between the frontal and right temporal lobes, frontal and parietal lobes, frontal and left temporal lobes, right temporal and left temporal lobes, right temporal and parietal lobes, left temporal and parietal lobes, right temporal and occipital lobes, parietal and occipital lobes, left temporal and occipital lobes, frontal and occipital lobes.

The protocol for BLT in patients with AD and their caregivers is shown in Figure 1. First, we recruited participants based on the inclusion and exclusion criteria. At baseline, all the participants were asked to complete assessments including neurophysiological scales (MMSE, ADAScog, and HAMD), EEG examination, and blood tests (i.e., Aβ, IL-1β, and MT levels). Subsequently, they were exposed to bright light at an illumination intensity of 14000 lux twice daily. After 4 weeks of therapy, the same assessments were repeated.

The characteristics of the patients included in this study are summarized in Table 1. In total, 22 patients with AD were included, with a mean age of 68.05 years, 10 men and 12 women. The caregiver group consisted of 23 individuals (8 men and 15 women) with a mean age of 65.04 years. Eleven patients with AD were taking memantine and nine were taking donepezil.

**TABLE 1:** Patients’ baseline characteristics.

Participant variables		AD (N=22)	HC (N=23)	*t*/χ^2^	*p* value
Age (years)		68.05 (4.24)	65.04 (8.72)	−1.479	0.149
Sex (M/F)		10/12	8/15	1.800	0.180
Education (years)		6.09 (1.34)	5.91 (1.44)	−0.428	0.671
BMI		22.49(3.33)	23.91(2.02)	1.718	0.095
MMSE (scores)		15.91 (7.51)	29.09 (1.13)	8.145	**0.000**
ADAScog (scores)		39.39 (19.45)	6.74 (2.84)	−7.793	**0.000**
HAMD (scores)		3.26(2.82)	7.27(4.61)	−3.502	**0.001**
Blood Aβ (ng/mL)		426.30(69.59)	306.38(83.63)	−5.216	**0.000**
Blood Aβ_40_ (pg/mL)		352.54 (86.61)	285.06 (80.86)	−2.703	**0.01**
Blood Aβ_42_ (pg/mL)		663.62 (107.39)	525.18 (117.47)	−4.121	**0.000**
Blood IL-1β (pg/mL)		87.37 (13.49)	63.73 (13.33)	−5.912	**0.000**
Blood MT (pg/mL)		7.54(2.11)	10.03 (2.63)	3.482	**0.001**
EEG power (encephalic region)					
	Frontal	0.29(0.10)	0.22 (0.04)	−3.285	**0.003**
	Left temporal	0.26 (0.07)	0.24 (0.05)	−1.161	0.252
	Right temporal	0.28(0.10)	0.21(0.04)	−2.902	**0.007**
	Parietal	0.25(0.07)	0.19(0.04)	−2.918	**0.006**
	Occipital	0.27(0.07)	0.22(0.05)	−2.569	**0.014**
EEG power (wave band)					
	Delta	2.76(0.89)	2.22(0.46)	−2.523	**0.017**
	Theta	1.64(0.75)	1.15(0.46)	−2.641	**0.012**
	Alpha	0.80(0.27)	0.58(0.12)	−3.677	**0.001**
	Beta	0.48(0.23)	0.42(0.12)	−1.022	0.312
Medications					
	Memantine	11	0	NA	NA
	Donepezil	9	0	NA	NA
	Antidepressant	12	0	NA	NA

Data are presented as mean(standard deviation, SD). AD, Alzheimer’s disease; HC, health control;MMSE, Mini-Mental State Examination; EEG, electroencephalography; PSD, power spectral density; NA, not applicable; HAMD, hamilton depression scale; ADAScog, Alzheimer’s Disease Assessment Scale—Cognitive Subscale; MT, melatonin; IL, interleukin; BMI, body mass index. Resting state EEG, power was determined during the closed eye state. Delta band, 0–4 Hz; Theta band, 4–8 Hz; Alpha band, 8–13 Hz; Beta band, 13–30 Hz. Where bold values represents *p* < 0.05.

### Effect of BLT on patients with AD and their caregivers

Blood test results and EEG parameters of patients with AD and their caregivers were evaluated. The results are presented in Table 2. After BLT, blood Aβ and IL-1β levels decreased significantly in both the AD and HC groups, whereas MT levels increased significantly in both groups. The HAMD score also decreased. Blood Aβ_40_ and Aβ_42_ levels were only significantly decreased in the HC group.

**TABLE 2:** Participants’characteristics pre- and post-bright light therapy.

Variables	AD (N = 22)		*t*/χ^2^	*p* value	HC (N = 23)		*t*/χ^2^	*p* value
	Pre	Post			Pre	Post		
Blood Aβ (ng/mL)	426.30(69.59)	380.27(60.64)	4.436	**0.000**	306.38(83.63)	235.79(59.63)	5.713	**0.000**
Blood Aβ_40_ (pg/mL)	352.54(86.61)	330.93(74.83)	1.417	0.171	285.06 (80.86)	199.63 (51.56)	4.185	**0.000**
Blood Aβ_42_ (pg/mL)	663.62(107.39)	693.82(107.56)	−1.634	0.117	525.18 (117.47)	406.26(81.20)	6.241	**0.000**
Blood IL-1β (pg/mL)	87.37(13.49)	77.54(12.41)	3.976	**0.001**	63.73(13.33)	50.65(9.54)	3.671	**0.001**
Blood MT (pg/mL)	7.54(2.11)	9.42(2.20)	−3.701	**0.001**	10.03(2.63)	14.02 (1.78)	−6.480	**0.000**
EEG power (encephalic region)								
Frontal	0.29 (0.10)	0.31 (0.11)	−0.513	0.611	0.22 (0.04)	0.24 (0.06)	−1.455	0.153
Left temporal	0.26 (0.07)	0.27 (0.08)	−0.483	0.632	0.24 (0.05)	0.26 (0.07)	−1.211	0.232
Right temporal	0.28 (0.08)	0.28 (0.10)	−0.164	0.870	0.21 (0.04)	0.22 (0.05)	−0.692	0.493
Parietal	0.25 (0.07)	0.26 (0.08)	−0.644	0.523	0.20 (0.04)	0.22 (0.04)	−1.693	0.098
Occipital	0.27 (0.07)	0.28 (0.08)	−0.343	0.734	0.22 (0.05)	0.25 (0.06)	−1.467	0.149
EEG power (wave band)								
Delta	2.76 (0.89)	2.75 (0.90)	0.038	0.970	2.22 (0.46)	2.35 (0.54)	−0.819	0.417
Theta	1.64 (0.75)	1.73 (0.76)	−0.394	0.695	1.15 (0.46)	1.18 (0.49)	−0.205	0.838
Alpha	0.80 (0.27)	0.83 (0.25)	−0.409	0.684	0.58 (0.12)	0.62 (0.15)	−1.162	0.251
Beta	0.48 (0.23)	0.51 (0.19)	−0.556	0.581	0.42 (0.12)	0.49 (0.16)	−1.795	0.080
HAMD(scores)	7.23(4.61)	6.00(4.47)	2.494	**0.021**	3.26 (2.82)	1.87 (2.51)	4.449	**0.000**

Data are presented as mean (standard deviation, SD). AD, Alzheimer’s disease; HC, health control; Aβ, β amyloid; MT, melatonin; EEG, electroencephalograph; HAMD, Hamilton depression scale. Resting state EEG, power was determined during the closed eye state. Delta band, 0–4 Hz; Theta band, 4–8 Hz; Alpha band, 8–13 Hz; Beta band, 13–30 Hz. Where bold values represents *p* < 0.05.

### Changes in EEG power after BLT

**FIGURE 2:**
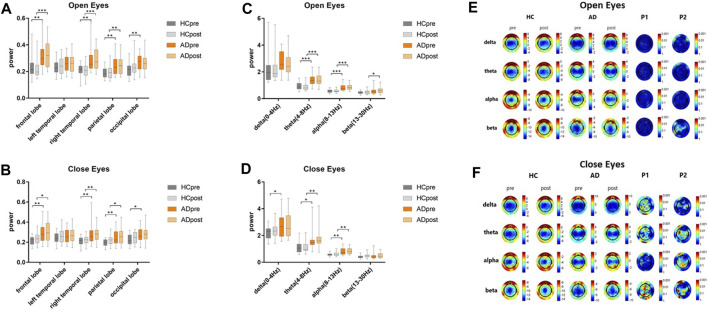
Resting state EEG power of patients with AD and their caregivers with normal cognition (CN group) in the open-eye **(A)** and closed-eye **(B)** states at the frontal, left temporal, right temporal, parietal, and occipital lobes, and in the open-eye **(C)** and closed-eye **(D)** states in the delta (0–4 Hz), theta (4–8 Hz), alpha (8–13 Hz), and beta (13–30 Hz) bands. The gray and orange bars represent the HC and AD groups, respectively before (dark color) and after (light color) bright light therapy. Resting state EEG topographic maps of patients with AD and their care givers with normal cognition (HC group) in the open-eye **(E)** and closed-eyes **(F)** states. P1 and P2 represent different *p* values before and after therapy in the HC and AD groups, respectively.

Resting state EEG was performed in patients with AD and their caregivers with normal cognitive levels, as shown in Figures 2A–D. Compared to people with normal cognition, patients with AD had higher resting state EEG power at baseline. In the open-eye state (Figure 2A), the power of the HC group decreased, whereas that of the AD group increased, although no significant change in the EEG power after BLT was observed in both groups compared with that at baseline,. BLT suppressed the resting state power in the occipital lobe of patients with AD and in the frontal and right temporal lobes of caregivers in the HC group; however, these results were not significant. Regarding the differences in EEG power between the AD and HC groups in the closed-eye state (Figure 2B), the differences after BLT decreased in frontal, parietal and occipital lobes compared with that before BLT.

Regarding the EEG power band, differences were observed at baseline between patients with AD and their caregivers in the theta, alpha, and beta bands in the open-eye state and in the delta, theta, and alpha bands in the closed-eye state. Although no significant changes were observed in the power bands of both the AD and CG groups after BLT, differences were observed between the beta band in the open-eye state and that of the theta band in the closed-eye state, whereas the differences between the delta bands in the closed-eye state changed from significant to not significant.

Regarding the EEG topographic map (Figures 2E, F) the difference between pre- and post-therapy in the HC and AD groups was not significant in the open-eye state. In the closed-eye state, the differences between the delta, theta, and beta bands of the occipital lobe in the HC group were significant (P1 < 0.001); the differences between the theta and beta bands of the left temporal parietal lobe in the AD group were also significant (P2 < 0.001).

**FIGURE 3:**
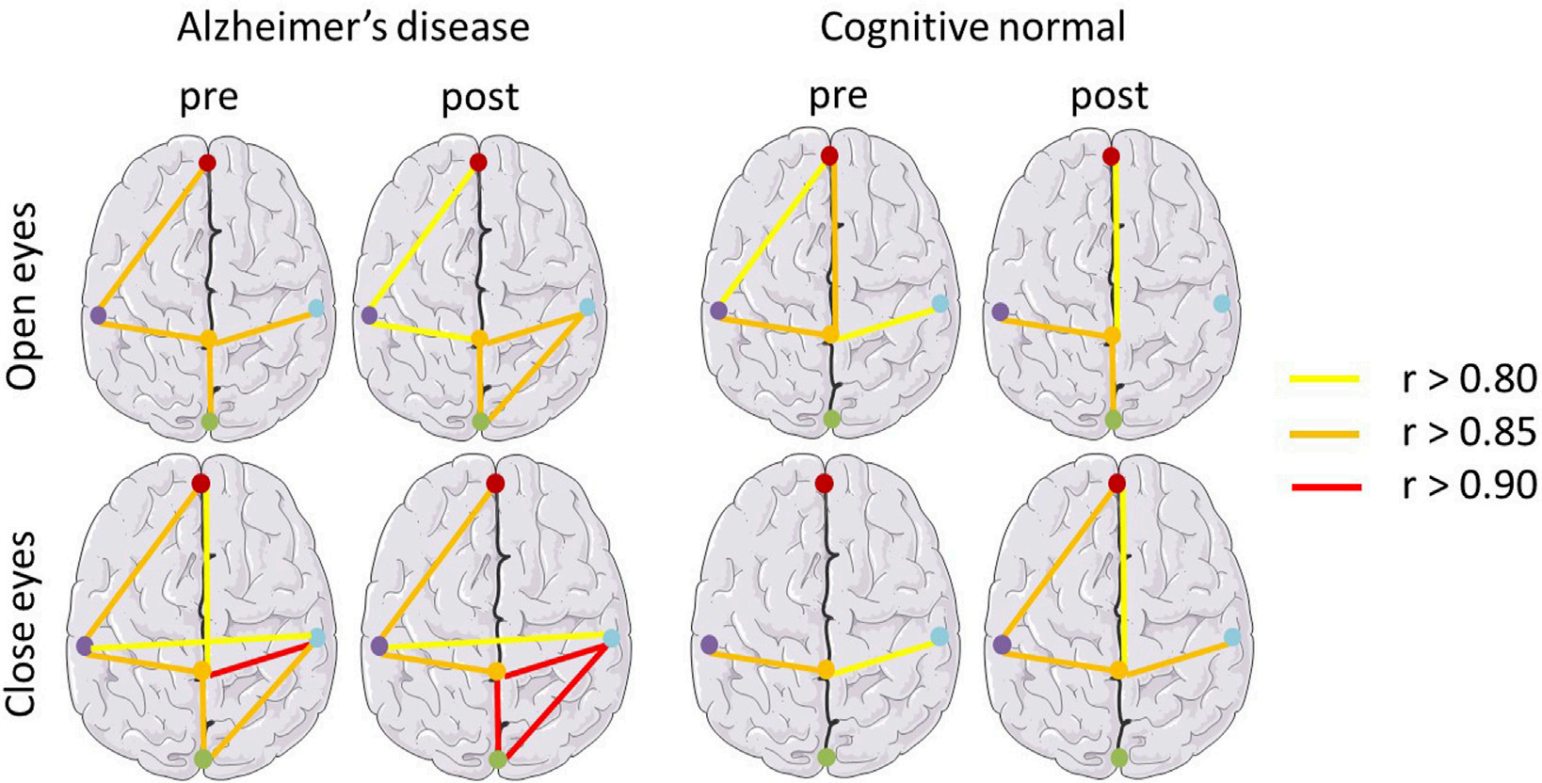
EEG power network of the brain in patients with Alzheimer’s disease and cognitively normal controls. Pearson coefficient (r) was used to represent the connection strength between regions of the cortex. r values greater than 0.80, 0.85, 0.90 are represented by yellow, orange and red bars respectively.

In Figure 3, the effects of BLT on the correlations between different regions of the cortex were investigated using the EEG power network in patients with AD and cognitive normal controls. The Pearson coefficient (r) was used to represent the connection strength between regions of the cortex as shown in Table 3. Compared with the pre therapy condition, significant improvements were observed in the numbers and strengths of connections after BLT.

**TABLE 3:** Correlation between regions of the cortex.

	AD_OE		AD_CE		HC_OE		HC_CE	
	pre	post	pre	post	pre	post	pre	post
①	0.869^***^	0.820^***^	0.876^***^	0.861^***^	0.813^***^	0.786^***^	0.733^***^	0.854^***^
②	0.726^***^	0.589^**^	0.805^***^	0.769^***^	0.885^***^	0.842^***^	0.754^***^	0.833^***^
③	0.597^**^	0.510^*^	0.798^***^	0.641^**^	0.714^***^	0.624^**^	0.634^**^	0.702^***^
④	0.710^***^	0.698^***^	0.831^***^	0.802^***^	0.755^***^	0.593^**^	0.743^***^	0.675^***^
⑤	0.854^***^	0.801^***^	0.855^***^	0.871^***^	0.865^***^	0.850^***^	0.852^***^	0.864^***^
⑥	0.865^***^	0.888^***^	0.941^***^	0.935^***^	0.847^***^	0.717^***^	0.845^***^	0.864^***^
⑦	0.641^**^	0.505^*^	0.686^***^	0.723^***^	0.772^***^	0.776^***^	0.762^***^	0.791^***^
⑧	0.855^***^	0.858^***^	0.889^***^	0.918^***^	0.769^***^	0.820^***^	0.723^***^	0.785^***^
⑨	0.784^***^	0.861^***^	0.891^***^	0.904^***^	0.776^***^	0.689^***^	0.700^***^	0.694^***^
⑩	0.496^*^	0.306	0.577^**^	0.570^**^	0.723^***^	0.725^***^	0.570^**^	0.759^***^

① to ⑩ represented relationship between frontal lobe and right temporal lobe, frontal lobe and parietal lobe, frontal lobe and left temporal lobe, right temporal lobe and left temporal lobe, right temporal lobe and parietal lobe, left temporal lobe and parietal lobe, right temporal lobe and occipital lobe, parietal lobe and occipital lobe, left temporal lobe and occipital lobe, frontal lobe and occipital lobe. *** *p* < 0.001, ** *p* < 0.01, * *p* < 0.05.

### Pearson correlation between cognitive, blood, and EEG parameters

**FIGURE 4:**
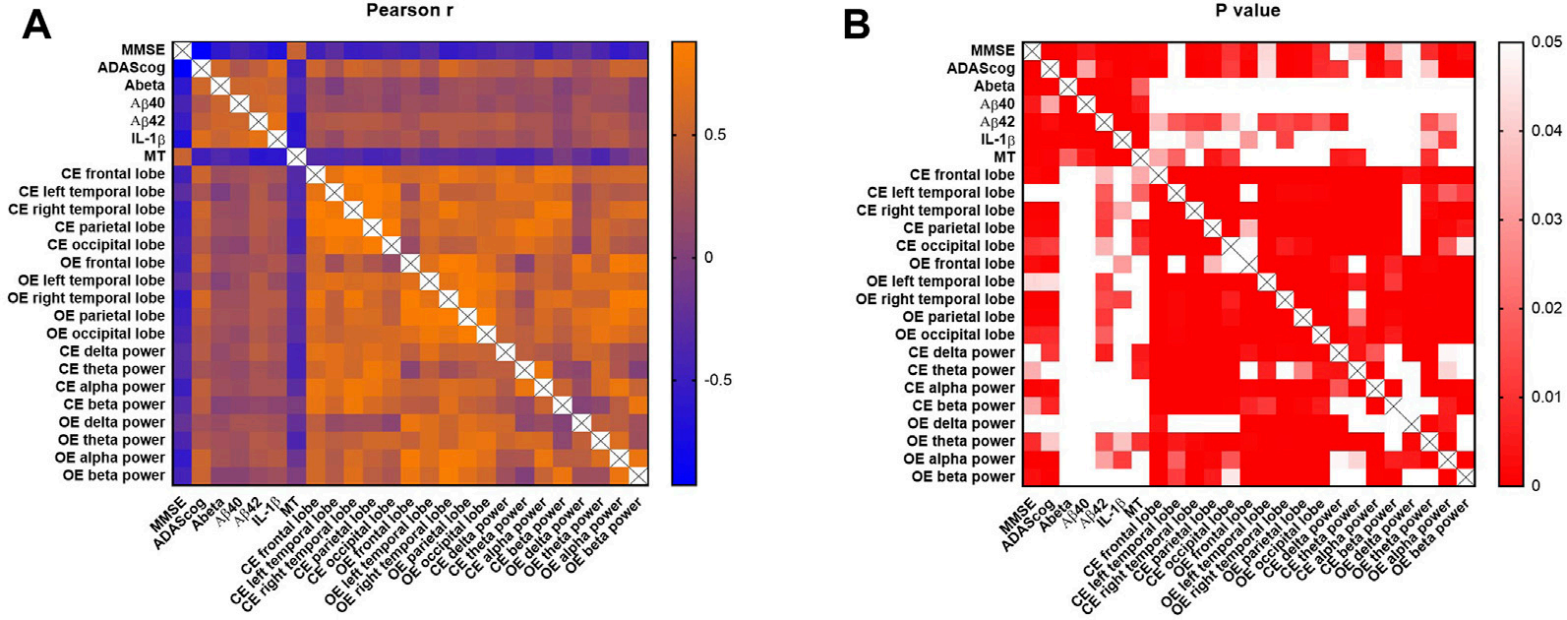
Pearson correlation analysis of cognitive, blood, and EEG parameters. MMSE and ADAScog scores represent the cognitive levels of participants. The blood parameters included Aβ, Aβ_40_, Aβ_42_, IL-1β and MT. EEG parameters included the power of five lobes (frontal, left temporal, right temporal, parietal, and occipital lobes) and four bands (delta [0–4 Hz], theta [4–8 Hz], alpha [8–13 Hz] and beta [13–30 Hz]). The scale bar represents the Pearson coefficient, r **(A)** and *p* values **(B)**. CE, closed-eye state; OE, open-eye state.

As shown in Figure 4, MMSE and ADAScog scores were both significantly correlated with the blood parameters of Aβ (r = −0.587, *p* < 0.001; r = 0.555, *p* < 0.001), Aβ_40_ (r = −0.410, *p* = 0.005; r = 0.319, *p* = 0.033), Aβ_42_ (r = −0.519, *p* < 0.001; r = 0.437, *p* = 0.003), IL-1β (r = −0.682, *p* < 0.001; r = 0.649, *p* < 0.001), and MT (r = 0.503, *p* < 0.001; r = −0.471, *p* = 0.001) respectively. The EEG parameter of the delta power band in the closed- (*p* = 0.054) and open-eye (*p* = 0.187) states showed no significant correlation with MMSE scores. The theta and delta power bands in the closed- (*p* = 0.096) and open-eye (*p* = 0.127) states showed no significant correlation with ADAScog scores. The EEG power of the left temporal lobe showed no significant correlation with both MMSE (*p* = 0.072) and ADAScog (*p* = 0.069) scores.

## Discussion

BLT is a medical treatment that utilizes natural or artificial light to improve a health condition. Our study showed that BLT may affect the EEG power and blood indicators, and may suppress depression, potentially owing to its effects on the circadian system *via* brain regions that respond preferentially to light. Patients with AD have high EEG power and strong electrical activity and more wastage in the resting state (Hsiao et al., 2013). Although the EEG power may be high and electrical activity of nerve cells may be strong in patients with AD, the synaptic function is incomplete and the signal conduction between neurons is abnormal; therefore, the brain cannot perform the functions of execution, memory, and learning (Babiloni et al., 2023).

To the best of our knowledge, EEG has been used as a non-invasive tool to study AD for many years. In recent years, modern methods of EEG analysis have been used to investigate physiological brain aging and AD (Cassani et al., 2018). Many recent studies focused on EEG signals for AD diagnosis, identifying and comparing key steps of EEG-based AD detection (Poil et al., 2013; Jesus et al., 2021). Resting state EEG was used as an effective tool to detect and assess cognitive impairment in patients with early AD (Meghdadi et al., 2021; Özbek et al., 2021). Compared to age-matched controls, an increase in spectral power was observed at the lower delta and theta frequencies in the AD group (Meghdadi et al., 2021), which was also observed in our study. The power of the delta and theta bands in the AD group decreased after BLT. These indicators may be potentially used as therapy efficacy assessment indicators.

In this study, the control group included cognitively normal individuals, rather than patients with AD who were not exposed to BLT. To some extent, this method avoided the placebo effect in participants with or without BLT. Moreover, the baseline values of the AD group were considered the self-control condition (positive control). The caregiver group was considered the normal condition (negative control). In previous studies, low intensity light was used as a sham stimulation (Hirakawa et al., 2020), which may be an ideal sham procedure if the lighting device is adjustable.

According to a previous study, non-invasive phototherapy is emerging as a strategy for suppressing Aβ self-assembly against AD (Ma et al., 2023). However, developing efficient photosensitizers for Aβ oxygenation that are active in deep brain tissues through the scalp and skull while reducing side effects remains a daunting challenge. Although light treatment improved cognitive function in AD mice and cleared amyloid levels in the brain (Yao et al., 2020; Kim et al., 2022), no changes in blood amyloid concentrations before and after BLT were observed. Another study found that BLT resulted in less daytime sleeping and increased night-time sleeping in people with dementia (Tan et al., 2022). Aβ levels can be cleared during sleep although accumulate during sleep deprivation (Shokri-Kojori et al., 2018; Wang and Holtzman, 2020).

Phototherapy is an effective method of treatment for patients with seasonal affective disorders (Knapen et al., 2014; Meyerhoff et al., 2018). Research confirms that phototherapy markedly improved mood and sleep quality in older adults (Zhang et al., 2023). Light signals are projected through retinal ganglion cells to depressed brain areas to participate in non-visual imaging functions, thereby activating nerve cell activity, secreting neurotransmitters to induce physiological changes in neural pathways, and regulating circadian rhythms, mood, and sleep in the biological organism to improve depressive symptoms (Fernandez et al., 2018). Moreover, the circadian system is composed of the central autonomous clock, suprachiasmatic nucleus, and body systems that follow the signals of the suprachiasmatic nucleus which mediate the effects of light on cognition and mood (Fernandez et al., 2018). It continuously changes the homeostatic set points of the body over the day-night cycle. The circadian rhythm is a natural, internal process that regulates the sleep-wake cycle that repeats approximately every 24 h.

Several treatments for mood disorders are available, including medication (anticonvulsants, antipsychotics, lithium, antidepressants, and benzodiazepines) and psychotherapy (Otte et al., 2016). Anticonvulsants, antipsychotics, and lithium are used to stabilize mood whereas antidepressants are used to treat depressive and anxiety disorders. As a non-pharmacological treatment, BLT can avoid the side effects and drug interactions associated with drug therapy, and also have the advantages of good compliance and ease of operation, making it suitable for use in patients with AD and their caregivers to treat depression.

This study has the following limitations and strengths. First, the sample size was relatively small. Regarding the long therapy duration and advanced age of participants, clinical samples were prone to shedding, and the dropout rate was high. The strength of this study is that we explored the non-pharmacological treatment BLT in patients with AD accompanied by depression, and revealed significant effects and useful clinical information regarding EEG power. Future studies will include a prolonged study duration and larger sample size to further investigate the association between changes in depression and EEG power.

## Conclusion

BLT can suppress depression in patients with AD and their caregivers. Meanwhile, BLT significantly affected the EEG power and blood indicators. Non-pharmacological antidepressant therapy may be an effective and safe method for improving the emotional states of patients with AD and their caregivers.

## Data Availability

The original contributions presented in the study are included in the article/Supplementary material, further inquiries can be directed to the corresponding author.
